# Practices of Rural Egyptian Birth Attendants During the Antenatal, Intrapartum and Early Neonatal Periods

**Published:** 2008-03

**Authors:** Gary L. Darmstadt, Mohamed Hassan Hussein, Peter J. Winch, Rachel A. Haws, Reginald Gipson, Mathuram Santosham

**Affiliations:** 1 Department of International Health, Bloomberg School of Public Health, Johns Hopkins University, 615 North Wolfe Street, Baltimore, MD 21205, USA; 2 Healthy Mother/Healthy Child Results Package, John Snow, Inc., Cairo, Egypt

**Keywords:** Home care, Community, *Daya*, Delivery, Newborn, Traditional birth attendant, Egypt

## Abstract

Neonatal deaths account for almost two-thirds of infant mortality worldwide; most deaths are preventable. Two-thirds of neonatal deaths occur during the first week of life, usually at home. While previous Egyptian studies have identified provider practices contributing to maternal mortality, none has focused on neonatal care. A survey of reported practices of birth attendants was administered. Chi-square tests were used for measuring the statistical significance of inter-regional differences. In total, 217 recently-delivered mothers in rural areas of three governorates were interviewed about antenatal, intrapartum and postnatal care they received. This study identified antenatal advice of birth attendants to mothers about neonatal care and routine intrapartum and postpartum practices. While mothers usually received antenatal care from physicians, traditional birth attendants (*dayas*) conducted most deliveries. Advice was rare, except for breastfeeding. Routine practices included hand-washing by attendants, sterile cord-cutting, prompt wrapping of newborns, and postnatal home visits. Suboptimal practices included lack of disinfection of delivery instruments, unhygienic cord care, lack of weighing of newborns, and lack of administration of eye prophylaxis or vitamin K. One-third of complicated deliveries occurred at home, commonly attended by relatives, and the umbilical cord was frequently pulled to hasten delivery of the placenta. In facilities, mothers reported frequent use of forceps, and asphyxiated neonates were often hung upside-down during resuscitation. Consequently, high rates of birth injuries were reported. Priority areas for behaviour change and future research to improve neonatal health outcomes were identified, specific to type of provider (physician, nurse, or *daya*) and regional variations in practices.

## INTRODUCTION

Child survival initiatives in Egypt have reduced the rate of mortality of children aged less than five years (under-five mortality) by half over the past 15 years, from 103 per 1,000 to 43.5 per 1,000 livebirths (1,2). Because infant and neonatal mortality have not followed the declines in child and maternal mortality in Egypt as elsewhere, they constitute an increasing proportion of under-five mortality. In 2000, infant mortality accounted for 80% of all under-five mortality; more than 40% of under-five deaths were neonatal deaths (2). A longitudinal study from 1994 to 1996 in two Egyptian governorates—Minya and Qaliubia—found that 80% of neonatal deaths occurred during the first week (3). Two-thirds of mothers whose neonates died noticed no obvious symptoms prior to the death of their babies, and care-seeking was rare, particularly during the first week. Throughout developing countries, neonatal deaths occur due primarily to infections, asphyxia, prematurity, and congenital anomalies and usually occur at home (4-6). Most deaths and adverse sequelae of complications are preventable with prompt and appropriate treatment and/or referral practices (5,7), emphasizing the importance of birth attendants as neonatal care providers.

Traditional birth attendants (*dayas*) attend 42% of deliveries in Egypt, and 60% of deliveries occur at home (1), suggesting that effective training and supervision of *dayas* in essential maternal and newborn care could have far-reaching effects. None of the five previous Egyptian studies on *daya* practices (8-11) focused on neonatal care, portraying the neonatal caregiving role of *dayas* as restricted to monitoring the umbilical stump for infection (12). However, reports suggest that *dayas* employ potentially harmful practices, such as administration of oxytocin and anaesthesia and use of forceps (9), practices unintentionally causing up to 12% of maternal deaths (8). Exactly how practices of *dayas* contribute to maternal and neonatal morbidity and mortality remains unknown.

Although *dayas* have not reduced maternal mortality, results of a study by Jokhio *et al*. in Pakistan and a meta-analysis by Sibley and Sipe suggest that *dayas* could be trained to provide routine care for mothers and newborns, particularly where skilled birth attendants are in a short supply, as in much of rural Egypt (7,13). *Dayas* could be trained in essential maternal care, including clean delivery; prompt recognition and referral of complications, including haemorrhage and sepsis danger signs; breastfeeding and nutrition counselling, including managing feeding problems; and avoidance of use of oxytocin, anaesthesia, or forceps. *Dayas* could also be trained in essential newborn care, potentially including recognition, management, and referral of birth asphyxia; identification and referral of neonates with low birthweight (LBW); promotion of early/exclusive breastfeeding; prevention and management of hypothermia; skin and umbilical cord care; and prompt recognition of and referral for danger signs. To identify training opportunities for *dayas* and other genres of birth attendants, this programmatic survey identified antenatal advice of birth attendants to mothers, routine intrapartum and postpartum practices, and potentially beneficial practice modifications.

## MATERIALS AND METHODS

Survey sites were selected based on the availability of project-trained community workers (*raedat*) in rural areas of the Aswan, Luxor, and Fayoum governorates, where the Healthy Mother/Healthy Child Results Package (HM/HCRP) of John Snow, Inc. (JSI), Cairo, Egypt, has worked. *Raedat* monitored all births and deaths among their assigned households. All women living in these areas who had delivered a live or dead newborn ≥7 days before the interview were included. In February 2000, eligible participants were approached by the *raedat,* who explained the study to them. No women refused to be interviewed. Participants provided their verbal informed consent prior to being interviewed about the antenatal, intrapartum and early postnatal care that they and their newborns received from the birth attendant. Permission for the survey was granted by the funding agency (United States Agency for International Development, USAID), the programme implementers (JSI), and the Egyptian Government.

The investigators devised a comprehensive questionnaire of birth attendant care practices to assess: (a) antenatal care, including provider, number of visits, and reason for visits; (b) peripartum maternal care, including infection control, recognition of danger signs, and postnatal visits, (c) intrapartum and immediate neonatal care, including resuscitation, care of the umbilical cord, assessment of size, thermal control, and skin care, and (d) postnatal neonatal care, including maternal advice and identification/management of danger signs. External consultants and Egyptian Government officials reviewed and revised the questionnaire. From the questionnaire, a relevant subset of questions was adapted, translated into Arabic, back-translated and checked for accuracy, and pretested before implementation. Face and content validity of the questionnaire was achieved through internal validating questions, pilot testing in the field, and reviewing and adapting the survey with *raedat* during training to assure that they knew how to ask the questions and that the questions conveyed the intended meaning.

All *raedat*, some of whom were nurses, had some high school education and prior interviewing experience. A one-day on-site training on study objectives, identification and recruitment of eligible women, and relevance of each question was conducted. Each *raedat* identified eligible women, visited households, and conducted interviews. *Raedat* interviewed as many as five women, but most interviewed one or two. Each interview took approximately 1–2 hour(s). In Fayoum, senior *raedat* supervised collection of data, and the Assistant Director for Maternal and Child Health Care Services monitored quality control. In Luxor and Aswan, peer-to-peer review of completed questionnaires ensured the quality of data. Supervisors facilitated prompt review of cases. For peer verification of data, identifiable data were available on questionnaires, but were masked after review. Data were manually validated using validating items in pre-analysis of the questionnaire; results below are from valid items. Percentages were based on total responses to each question. The statistical significance of differences observed between regions was computed using the chi-square tests.

## RESULTS

### Study population

Copies of the questionnaires were administered at home to 217 mothers in the Fayoum, Luxor, and Aswan governorates ≥7 days after birth. Seventy-six percent of mothers were aged 20–34 years. Sixty percent were from Fayoum, 24% were from Luxor, and 16% were from Aswan.

### Antenatal care

In the majority (>70%) of cases, physicians provided antenatal care (Table 1). In Aswan and Luxor, most mothers (88%, 30/34; and 83%, 44/53 respectively) visited a physician at least once, four times more often than in Fayoum (p<0.001). Twenty percent of women visited a nurse for antenatal care in Fayoum, but almost none visited a nurse in Aswan or Luxor (Table 1). *Dayas* rarely provided antenatal care, seeing only three mothers, exclusively in Fayoum (Table 1). While antenatal care attendance varied, most women had at least one antenatal care visit, and one-third of mothers had complete antenatal care (≥4 visits) (Table 1). Antenatal care attendance varied by region: in Aswan, most (82%) mothers had complete antenatal care, but in Fayoum, only 16% of mothers had complete antenatal care. Complete antenatal care attendance was significantly higher in Aswan than in Luxor (p<0.001) or Fayoum (p<0.001).

**Table 1:** Selected survey results

Reported care received	Fayoum (n=130*)	Luxor (n=53*)	Aswan (n=34*)	Overall (n=217*)
Antenatal care				
Attendance				
Any antenatal care	101/123 (82)	44/53 (83)	32/34 (94)	177/211 (84)
Complete antenatal care	20/123 (16)	21/53 (40)	28/34 (82)	70/211 (33)
Provider				
From physician	54/126 (43)	44/53 (83)	30/34 (88)	128/213 (60)
From nurse	24/126 (19)	0/53 (0)	2/34 (6)	26/213 (12)
From *daya*	3/126 (2)	0/53 (0)	0/34 (0)	3/213 (1)
Intrapartum care				
Birth attendant				
Obstetrician	19/124 (15)	13/53 (25)	21/34 (62)	55/211 (26)
General practitioner	2/124 (2)	9/53 (17)	7/34 (21)	18/211 (9)
Nurse	20/124 (16)	0/53 (0)	2/34 (6)	22/211 (10)
*Daya*	72/124 (58)	16/53 (30)	3/34 (9)	91/211 (43)
Relatives/other	5/124 (4)	15/53 (28)	1/34 (3)	21/211 (10)
Clean delivery				
Washed hands before examination/delivery	80/122 (66)	45/53 (85)	27/34 (79)	152/209 (73)
If washed hands, used soap	50/80 (63)	18/37 (49)	6/27 (22)	74/144 (51)
If washed hands, used alcohol	24/80 (30)	18/37 (49)	17/27 (63)	59/144 (41)
Cleaned delivery instruments	64/130 (49)	27/53 (51)	21/34 (62)	112/217 (52)
Site of complicated deliveries				
Home	13/28 (46)	1/7 (14)	0/8 (0)	14/43 (33)
Private clinic/hospital	8/28 (29)	4/7 (57)	2/8 (25)	14/43 (33)
Public clinic/hospital	7/28 (25)	2/7 (29)	6/8 (75)	15/43 (35)
Use of forceps				
Obstetrician	10/16 (63)	4/5 (80)	7/8 (88)	21/29 (72)
General practitioner	3/16 (19)	1/5 (20)	1/5 (20)	5/29 (17)
Nurse	0/16 (0)	0/5 (0)	0/8 (0)	0/29 (0)
*Daya*	2/16 (13)	0/5 (0)	0/8 (0)	2/29 (7)
Advice				
Breastfeeding	86/130 (66)	28/53 (53)	27/34 (79)	141/217 (65)
Colostrum	90/130 (69)	19/53 (36)	27/34 (79)	136/217 (63)
Cord care	77/130 (59)	6/53 (11)	17/34 (50)	100/217 (46)
Skin care	47/130 (36)	8/53 (15)	3/34 (9)	58/217 (27)
Warmth provision	66/130 (51)	3/53 (6)	14/34 (41)	83/217 (38)
Newborn hygiene	65/130 (50)	9/53 (17)	18/34 (53)	92/217 (42)
Recognition of danger signs	37/130 (28)	1/53 (2)	8/34 (24)	46/217 (21)
Other				
Delay >1 hour in mother-newborn contact	30/130 (23)	12/53 (23)	12/34 (35)	126/217 (58)
Pulled cord to deliver the placenta	13/130 (10)	4/53 (8)	7/34 (21)	24/217 (11)
Birth injury	5/115 (4)	4/49 (8)	5/30 (17)	14/194 (7)
Limb problems	1/116 (1)	4/50 (8)	0/33 (0)	5/199 (3)
Postnatal care				
Umbilical cord care				
Sterile cord-cutting	57/92 (62)	37/47 (79)	31/32 (97)	125/171 (73)
Implement used for cutting cord				
New home razor	35/127 (28)	12/50 (24)	1/33 (3)	48/210 (23)
Used home razor	2/127 (2)	0/50 (0)	0/33 (0)	2/210 (1)
Attendant's razor	22/127 (17)	6/50 (12)	1/33 (3)	29/210 (14)
Attendant's scissors	64/127 (50)	32/50 (64)	30/33 (91)	126/210 (60)
Other	4/127 (3)	0/50 (0)	1/33 (3)	5/210 (2)
Sterile cord tying	55/95 (58)	31/40 (78)	33/34 (97)	119/169 (70)
Material used to tie off cord				
Attendant's clamp	29/126 (23)	3/47 (6)	24/33 (73)	56/206 (27)
Clamp from home	1/126 (1)	0/47 (0)	2/33 (6)	3/206 (1)
Attendant's thread	61/126 (48)	30/47 (64)	4/33 (12)	95/206 (46)
Thread from home	34/126 (27)	13/47 (28)	2/33 (6)	49/206 (24)
Other	1/126 (0)	1/47 (2)	1/33 (3)	3/206 (1)
Substance applied to cord				
Alcohol	68/124 (55)	17/50 (34)	9/34 (26)	94/208 (45)
Other disinfectant	0/124 (0)	9/50 (18)	7/34 (21)	16/208 (8)
Other	17/124 (14)	21/50 (42)	9/34 (26)	47/208 (23)
Nothing	39/124 (34)	3/50 (6)	9/34 (26)	51/208 (25)
Birth asphyxia intervention given	31/129 (24)	21/53 (40)	12/34 (35)	64/216 (30)
Newborn hung by legs	3/31 (10)	2/21 (10)	4/12 (33)	9/64 (14)
Mouth-to-mouth	4/31 (13)	2/21 (10)	0/12 (0)	6/64 (9)
Newborn weighed	20/119 (17)	4/50 (8)	10/34 (29)	34/203 (17)
Thermal control				
Delivery room warm	96/126 (76)	38/52 (73)	13/30 (43)	147/208 (71)
Dried/wrapped promptly	116/118 (98)	48/52 (92)	34/34 (100)	202/208 (97)
Baby felt cold	24/106 (23)	9/50 (18)	8/32 (25)	41/208 (20)
Postnatal visit by birth attendant	100/117 (85)	20/43 (47)	22/34 (65)	142/194 (73)

*The number of respondents may be smaller than the total number, depending on total responding to a given question

Figures in parentheses indicate percentages

### Intrapartum care

Approximately 62% (133/214) of mothers reported uncomplicated deliveries. More than one-third considered their delivery longer (22%, 46/214), more difficult (10%, 21/214), or more problematic (7%, 14/214) than expected. Among the deliveries mothers considered complicated, the delivery was as likely to take place in the home or in a private clinic as a public hospital (Table 1). Delivery of a complicated birth at home was most likely in Fayoum (Table 1).

The use of forceps was common, usually by obstetricians but sometimes by general practitioners (Table 1). In deliveries by obstetricians, forceps were used 38% (21/55) of the time and in more than half of deliveries (10/16) in Fayoum. The general practitioners used forceps less than obstetricians (Table 1). While no nurses used forceps, two *dayas* in Fayoum used them (Table 1). Some mothers reported that the umbilical cord was pulled to deliver the placenta; birth injury including face scratches and body bruises, particularly in Aswan; and limb problems, predominantly in Luxor (Table 1).

*Dayas* attended roughly half of deliveries and were more common birth attendants than physicians, nurses, or relatives (Table 1). Type of birth attendant varied by region: in Fayoum, *dayas* were much more common than in Luxor or Aswan (Table 1). This was reversed for physicians, often obstetricians, who attended most births in Aswan, almost half in Luxor, and just 17% in Fayoum (Table 1). Nurses attended 16% (20/124) of deliveries in Fayoum and 6% (2/34) in Aswan, but none in Luxor. Untrained relatives occasionally served as birth attendants in Luxor and Fayoum, but attended almost one-third of births in Aswan (Table 1).

### Hygiene and cord care

In most cases, birth attendants practised hand-washing before vaginal examinations or delivery, usually with soap and water or alcohol (Table 1). In half of cases, birth attendants disinfected delivery instruments (Table 1) by boiling in water (29%, 62/217) or soaking in alcohol (23%, 50/217). Plain water was used in 12% (27/217) of cases.

Birth attendants usually used scissors, or occasionally a new razor, to cut the cord (Table 1). While a clamp brought by the birth attendant was sometimes used for tying off the cord, it was usually tied with thread brought by the birth attendant or found in the home. Mothers who noticed whether implements used to cutting/tying the cord were sterile believed that the implement was sterile in about three-quarters of cases (Table 1). In Aswan, birth attendants almost universally practised aseptic cutting and tying the cord, often using their own shears and clamps (Table 1). Antiseptic, usually alcohol, was used on the cord stump in half of neonates; lack of cord cleansing was most common in Fayoum and Aswan and was rare in Luxor (Table 1).

### Resuscitation

Seventy percent of newborns required no stimulation to cry. The most common resuscitative procedure was tapping on the back, performed in 45% (29/64) of resuscitative interventions (Fig). Mouth-to-mouth breathing was rarely performed (Table 1). Of all newborns needing resuscitation, 14% were hung by their legs; in Aswan, this figure was 33% (Table 1).

**Fig.**
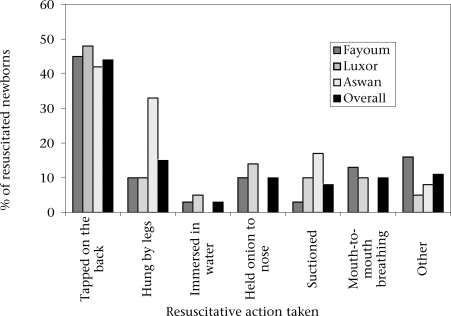
Reported resuscitation practices for birth asphyxia

### Assessment of birthweight

Most mothers felt that their baby arrived on time and was a sufficient birthweight, but 7% considered their baby preterm, and 11% believed that their baby was LBW. However, few birth attendants and no *dayas* weighed newborns (Table 1). Birth attendants rarely commented on size of newborns (4%, 8/198). Of babies whose mothers recalled their birthweight, 16% (4/25) were reportedly LBW (<2,500 g).

### Thermal control

The delivery room was usually warm, and nearly all neonates were wrapped soon after birth (Table 1). The *daya* (32%, 65/208), nurse (25%, 52/208), or maternal grandmother (19%, 40/208) usually covered the baby. In Aswan, the nurse generally performed this job (56%, 19/34), often to assist a physician. Still, 20% of mothers believed that their baby felt cold after delivery (Table 1).

### Health advice

Varying percentages (19–65%, 41–141/217) of mothers reported receiving specific neonatal care advice from birth attendants. Women in Luxor were much less likely to receive advice than those in Fayoum or Aswan. Half (50%, 108/217) of the mothers reported receiving some guidance about newborn care during the antenatal care visit. Two-thirds were counselled by a birth attendant to breastfeed and give their babies colostrum. Approximately half of the mothers received advice about care of umbilical stump, keeping the baby warm, and hygiene of newborns. However, fewer mentioned being told about neonatal bathing, skin care, infections, and danger signs (Table 1).

### Postnatal care

Birth attendants made a postnatal visit to the home of three-fourths of neonates within the first week of life (Table 1). Most newborns were visited on the first (17%), second (34%), or third (25%) days of life.

### Analysis

Results of the survey revealed variations in practices of birth attendants during the antenatal and peripartum periods. This study identified some commendable antenatal and delivery-care practices (Table 2), including high antenatal care attendance, routine hand-washing, sterile care of the umbilical cord, prompt thermal control, and postnatal visitation by birth attendants. These interventions can save lives and improve the health of newborns (5,14). However, this study also identified several suboptimal care practices (Table 2).

**Table 2:** Standard and suboptimal practices of reported birth attendants

Type of care	Standard practices	Suboptimal practices
Antenatal care	High overall antenatal care attendance	Low complete antenatal care attendance
Type of birth attendant		Relatives attending births, especially in Luxor
Hygiene/clean delivery	Birth attendants washed hands before 75% of vaginal examinations and deliveries Sterile technique in cutting/tying cord	Delivery instruments cleaned before use in only half of cases Unsterile instrument used for cutting and household thread to tie umbilical cord No antiseptic on cord stump
Intrapartum and immediate newborn care		Complications delivered at home and private clinics High rate of use of forceps by obstetricians Use of forceps by *dayas* Pulling umbilical cord to facilitate delivery of the placenta Hanging by legs during resuscitation
Thermal control	Prompt thermal control postdelivery	Low skin-to-skin contact Delay in initial mother-newborn contact (>1 hour in half of cases), especially in Aswan High rate of hypothermia
Postnatal care	75% had postnatal visit with birth attendant	
Care of high-risk newborns		Under-identification of low birthweight Lack of weighing
		Lack of communication with the mother about size of newborn
Prevention		Birth injury, especially in Aswan (17%), limb problems in Luxor (8%)

### Antenatal care

Antenatal care was provided commonly by physicians and rarely by *dayas*, corroborating the findings of Stanton and Langsten and the 2005 EDHS (69% by physicians vs 23% by *dayas*) (1,9). While nurses provided more antenatal care in Fayoum than anticipated, they were uninvolved in Luxor and Aswan. Overall, the coverage of antenatal care was good; 80% of mothers had ≥1 visit, and 30% had complete antenatal care. These data corroborated the findings of other studies (1,3,15), although the complete coverage of antenatal care in this study was lower than the Egypt Demographic and Health Survey (EDHS) estimate of 54% in rural areas. Overall, the data suggest that any improvements in quality of antenatal care require training of physicians (and training of nurses in Fayoum).

### Intrapartum care

Like Stanton and Langsten, this study found *dayas* to be more common than physicians as attendants at delivery (3), the converse of the 2005 EDHS estimate showing greater prominence of physicians than *dayas* (59.4% and 30.5% of rural births respectively) (1). The finding that relatives were involved in 13% of deliveries was much higher than the 2005 EDHS for rural births (2.3%) and is comparable with the findings of Stanton and Langsten (8%) (3). Attendance of nurses at 10% of deliveries, agreed with the findings (12%) of Stanton and Langsten and the EDHS (6.4%) (1,3). Type of delivery attendant varied by region (primarily obstetricians in Aswan vs *dayas* in Fayoum); this should be considered in targeting birth attendants for training. Complicated deliveries were as likely to occur at home or private clinics as in government hospitals, although deliveries were most common at home in Fayoum, at private clinics in Luxor, and at public hospitals in Aswan (Table 1). Further analysis is needed to determine the proportion of complicated deliveries beginning at home but moved to hospital.

### Advice

Postnatal visits provide important opportunities to provide care and advice regarding newborn care (5,10). High rates of postpartum visits in all the three governorates are promising and concur with the findings of previous studies (9), although their limited scope (inspection of the umbilical stump) suggests a missed opportunity for birth attendants to provide postnatal care and advice.

### Modifiable practices

This study identified several intrapartum practices that, if modified, could improve health outcomes of newborns. Relatives attended 28% of deliveries in Luxor; addressing cultural and infrastructural limits to the use of skilled birth attendants during delivery could improve the management of complications and care of newborns. Clean delivery could also be improved: in half of all cases, delivery instruments were not disinfected, and aseptic care of the umbilical cord was lacking. The use of forceps by obstetricians (nearly 40%) was high, particularly in Fayoum (>50%), both by Western standards and a previous Egyptian study (13%) (3).

Delays in initial mother-newborn contact and maternal reports of neonatal hypothermia suggest the need for training on thermal control, as has been reported for a number of other countries (16). Birth injuries were common, especially in Aswan, which had the highest proportion of babies delivered by obstetricians, direct observation of deliveries and warnings during training of birth attendants about potential harm from interventions to accelerate delivery may reduce such injuries.

As reported previously, few neonates were given advanced resuscitative measures, such as mouth-to-mouth breathing (17). However, as the incidence of birth asphyxia in the community in Egypt is unknown, many neonates might have benefited from more aggressive intervention than they received. Of those receiving an intervention for birth asphyxia, many were managed ineffectively, such as hanging the baby upside-down by the legs, another area for improving skills of birth attendants.

### High-risk neonates

LBW is a very important predictor of poor neonatal outcome, yet newborn weighing by birth attendants is rare (3). Despite the lack of communication about birthweight, mothers of weighed neonates could recall birthweight of the infant in 75% of cases. Training of birth attendants should emphasize the importance of recognizing high-risk LBW newborns and making appropriate referrals. Moreover, educating mothers about implications of birthweight could improve care-seeking for LBW babies.

High rates of reported hypothermia, coupled with reports that physicians and *dayas* rarely wrapped newborns after delivery, suggest that improved newborn thermal-control practices are needed, particularly for LBW/preterm infants.

## DISCUSSION

In addition to ongoing efforts to improve the quality of antenatal, intrapartum and postnatal care provided by skilled birth attendants, such as doctors and nurses, *dayas* are such common care providers that they should also be considered neonatal care programme resources in rural Egypt. The time *dayas* spend with mothers suggests an opportunity for an expanded role. Langsten found that practices of *dayas*, such as using sterilized scissors for cord-cutting, generally improved following training (9). He also found a two-fold increase in *dayas* who provided advice about hygiene, breastfeeding, warmth, and immunization (9). Other developing-country studies have shown similar improvements in post-training (13). While promising, upgrading skills of *dayas* is controversial. Certainly, economic development may lead Egyptian women to choose skilled prenatal and intrapartum care over *dayas* (9), and *dayas* currently view themselves only as delivery attendants. Stanton and Langsten found that 25% of *dayas* and fewer women in the community thought expectant mothers should see a *daya* antenatally (9,10). Still, *dayas* are prominent and widely accepted care providers for the large share of births at home in rural Egypt, suggesting that expanding their skills to provide intrapartum care and antenatal and postnatal advice to recognize and encourage care-seeking for complications could improve neonatal outcomes (9).

The importance of designing appropriate training curricula, monitoring impact, and providing ongoing supervision for *dayas* is illustrated by use of forceps among *dayas*, in this study and others (3). Stanton and Langsten found that all *dayas* who used forceps and other interventions to speed delivery had been trained through the Egypt Child Survival Project, suggesting inadequate post-training monitoring and supervision.

These findings of the survey have implications for the design of training programmes for birth attendants (Table 3). Specifically, such programmes should encourage appropriate infection control practices, disinfection of delivery instruments, and aseptic care of the cord. *Dayas* can be trained to monitor labour, to avoid interventions that speed delivery, and to provide effective resuscitation. Additionally, *dayas* can be trained to identify high-risk preterm and LBW newborns. During the postpartum period, *dayas* can be trained to encourage immediate mother-newborn contact, which prevents hypothermia and encourages initiation of breastfeeding; proper knowledge of skin-to-skin positioning can enhance these practices.

**Table 3:** Priority areas for future research

Antenatal care
Confirm content of routine newborn care advice to mothers
Determine acceptable ways to increase antenatal care attendance
Intrapartum care
Determine reasons complicated deliveries occur at home or in private clinics, and barriers to use of quality healthcare facilities
Audit use of forceps by birth attendants
Evaluate recognition of birth asphyxia by birth attendants and resuscitation techniques
Postnatal care
Evaluate thermal control knowledge/practice of birth attendants and determine causes of neonatal hypothermia
Determine reasons for delayed mother-newborn contact, lack of skin-to-skin contact, and delayed initiation of breastfeeding
Danger signs
Evaluate ability of birth attendants to recognize neonatal danger signs
Evaluate ability of birth attendants to use simple algorithms to identify serious illness
Evaluate referral practices of birth attendants
Overall
Determine cultural, socioeconomic, and infrastructural reasons for regional differences in practices
Quantify impact of birth attendants on neonatal morbidity and mortality

Unfortunately, insufficient data on mortality are available from this survey to assess the impact of practices of birth attendants on outcomes. Langsten found that interventions to speed delivery were not associated with increased neonatal mortality (3). The impact of these procedures on neonatal mortality was not disaggregated for *dayas*, nurses, and physicians, however, and requires further investigation (Table 3).

Relying on maternal memory of peripartum events to assess practices of birth attendants is prone to recall bias, although this was minimized by administering questionnaires shortly after delivery. The quantitative survey did not investigate the rationale for practices; thus, additional qualitative information is needed before designing optimally effective training strategies. While this study had limited geographic representation and sample size because it was restricted to programmatic sites, on average, care practices correlated with previous Egyptian and developing-country studies. However, the important regional differences captured suggest that Egyptian maternal and newborn health policies and programmes should be tailored for particular regions and developed based on quality data.

This article presents the most comprehensive examination of practices of birth attendants in rural Egypt to date. Significant regional variations observed in practices may be due to complex differences in the availability and quality of facilities, economic development indicators, and cultural variations. Further research is needed to understand the richness of these differences to improve training programmes for birth attendants. This and future studies may guide more effective allocation of resources. Appropriate training and supervision for birth attendants and investments in priority research areas (Table 3) could improve neonatal outcomes among Egyptian neonates and serve as a model for other countries in the region.

## ACKNOWLEDGEMENTS

This study was supported by the United States Agency for International Development through the Healthy Mother/Healthy Child Results Package Grant to John Snow, Inc., Cairo, Egypt. The authors thank Dr. Mohsen Lamia and Dr. Mohsen El-Said of HM/HCRP for their role in designing this study.

## References

[1] El-Zanaty F, Way A (2006). Egypt demographic and health survey, 2005.

[2] El-Zanaty F, Way AA (2001). Egypt demographic and health survey, 2000.

[3] Stanton B, Langsten R (1998). Rates of and factors associated with morbidity and mortality among Egyptian neonates and infants: a longitudinal prenatal and postnatal study.

[4] Lawn J, Cousens S, Zupan J (2005). Four million neonatal deaths: where? when? why? Neonatal survival series paper 1. Lancet.

[5] Darmstadt G, Cousens S, Adam T, Walker N, de Bernis L (2005). Evidence-based, cost-effective interventions: how many newborn babies can we save?. Lancet.

[6] Black R, Kelley L (1999). Reducing perinatal and neonatal mortality. Child Health Res Proj Spec Rep.

[7] Bhutta Z, Darmstadt GL, Hasan B, Haws R (2005). Community-based interventions for improving perinatal and neonatal outcomes in developing countries: a review of the evidence. Pediatrics.

[8] (1994). Child Survival Project. National maternal mortality study: findings and conclusions.

[9] Langsten R (1998). The role of traditional birth attendants in pre- intra- and post-natal care in Egypt: implications for training and program implementation.

[10] (1989). Social Planning, Analysis and Administration Consultants. *Daya*'s practices and maternal mortality in Giza, Egypt.

[11] (1999). United Nations Children's Fund. UNICEF *daya* training needs assessment.

[12] (1998). Social Planning, Analysis and Administration Consultants. SPAAC diagnostic study conducted for the MotherCare Project.

[13] Sibley L, Sipe T (2004). What can a meta-analysis tell us about traditional birth attendant training and pregnancy outcomes?. Midwifery.

[14] Jokhio A, Winter H, Cheng K (2005). An intervention involving traditional birth attendants and perinatal and maternal mortality in Pakistan. N Engl J Med.

[15] Yount K, Mechael P, Langsten R (1998). Perinatal/neonatal mortality and morbidity study. Results of the quantitative component.

[16] Dragovich D, Tamburlini G, Alisjahbana A, Kambarami R, Karagulova J, Lincetto O (1997). Thermal control of the newborn: knowledge and practice of health professionals in seven countries. Acta Paediatr.

[17] Saugstad OD, Rootwelt T, Aalen O (1998). Resuscitation of asphyxiated newborn infants with room air or oxygen: an international controlled trial: the Resair 2 study. Pediatrics.

